# Toward a Multivariate Prediction Model of Pharmacological Treatment for Women With Gestational Diabetes Mellitus: Algorithm Development and Validation

**DOI:** 10.2196/21435

**Published:** 2021-03-10

**Authors:** Carmelo Velardo, David Clifton, Steven Hamblin, Rabia Khan, Lionel Tarassenko, Lucy Mackillop

**Affiliations:** 1 Sensyne Health, plc Oxford United Kingdom; 2 Department of Engineering Science University of Oxford Oxford United Kingdom; 3 Oxford University Hospitals NHS Foundation Trust Oxford United Kingdom; 4 Nuffield Department of Women’s Reproductive Health University of Oxford Oxford United Kingdom

**Keywords:** gestational diabetes mellitus, mobile health, machine learning, algorithms

## Abstract

**Background:**

Successful management of gestational diabetes mellitus (GDM) reduces the risk of morbidity in women and newborns. A woman’s blood glucose readings and risk factors are used by clinical staff to make decisions regarding the initiation of pharmacological treatment in women with GDM. Mobile health (mHealth) solutions allow the real-time follow-up of women with GDM and allow timely treatment and management. Machine learning offers the opportunity to quickly analyze large quantities of data to automatically flag women at risk of requiring pharmacological treatment.

**Objective:**

The aim of this study is to assess whether data collected through an mHealth system can be analyzed to automatically evaluate the switch to pharmacological treatment from diet-based management of GDM.

**Methods:**

We collected data from 3029 patients to design a machine learning model that can identify when a woman with GDM needs to switch to medications (insulin or metformin) by analyzing the data related to blood glucose and other risk factors.

**Results:**

Through the analysis of 411,785 blood glucose readings, we designed a machine learning model that can predict the timing of initiation of pharmacological treatment. After 100 experimental repetitions, we obtained an average area under the receiver operating characteristic curve of 0.80 (SD 0.02) and an algorithm that allows the flexibility of setting the operating point rather than relying on a static heuristic method, which is currently used in clinical practice.

**Conclusions:**

Using real-time data collected via an mHealth system may further improve the timeliness of the intervention and potentially improve patient care. Further real-time clinical testing will enable the validation of our algorithm using real-world data.

## Introduction

### Background

Gestational diabetes mellitus (GDM), defined as glucose intolerance with onset or first recognition during pregnancy [1], increases the risk of morbidity in women and newborns. Successful management of GDM reduces maternal hyperglycemia and perinatal morbidity. Having a pregnancy complicated by GDM is associated with a risk of developing type 2 diabetes in the long term for women [2] and, later in life, for the offspring [3]. Standard clinical management for GDM is lifestyle advice and pharmacological treatment [4,5].

The increased prevalence of GDM [6], combined with limited resources available to the National Health Service (NHS) [7], is challenging the optimal delivery of care to women with GDM in the NHS.

GDM is usually diagnosed after 24 weeks of pregnancy, providing only a short intervention period (typically around 10 weeks) to influence perinatal outcomes. Therefore, timely identification of the need for pharmacological treatment is very important.

In current clinical practice, blood glucose (BG) data are provided by women in the form of paper-based diaries that are bought to clinics for clinician review. Decisions regarding GDM management therefore occur only during these reviews.

### Benefits of Mobile Health

Access to real-time data recorded in electronic diaries could enable *between-clinic* recognition of patterns in the data and allow midwives to adjust medication in a timely fashion so that women have more chance of tighter control of their BG readings, facilitating improved clinical outcomes for the woman and her baby.

Mobile health (mHealth; ie, internet-linked mobile devices to monitor patients’ health) is well placed to facilitate this type of care and provide health care providers access to a larger and richer set of data on which to base their clinical decisions [8].

The use of mHealth by women with GDM can simplify their daily routine and has been shown to provide an easy and acceptable way of collecting, storing, and analyzing their data to aid personal decision making [9,10].

Digitally monitored BG can also provide additional benefits to clinicians by enabling real-time reviews and customized feedback. Furthermore, using the collected data could lead to the development of algorithms for the early identification of the need for pharmacological treatment, allowing earlier intervention, more frequent reviews, and potentially improved outcomes.

Some studies have successfully used baseline characteristics to predict the need for pharmacological treatment or maternal outcomes. Among these studies, factors such as glucose tolerance test results, maternal age, maternal BMI, ethnicity, and previous pregnancy history were identified as predictors of the requirement for pharmacological therapy [11-16].

### Prediction of Pharmacological Treatment

Barnes et al [12] analyzed a cohort of 3317 women with GDM to predict pharmacological treatment using variables from electronic patient records. The authors validated their model using data from different time periods (eg, 1992-2004 and 2005-2015) and multiple clinics (Bankstown-Lidcombe Hospital Diabetes Centre and Liverpool Hospital Diabetes Centre). Their algorithm was capable of a positive predictive value of 87.6%, negative predictive value of 69.9%, sensitivity of 93%, specificity of 99.4%, and the area under the receiver operating characteristic curve (AUC) value of 0.712 (95% CI 0.693-0.731). This study makes use of variables at diagnosis, such as the oral glucose tolerance test and glycated hemoglobin (HbA_1c_) results, to predict the need for pharmacological treatment. In contrast to our study, the authors do not include operational, real-time BG readings, which may be fundamental to obtain better predictions.

**Figure 1:**
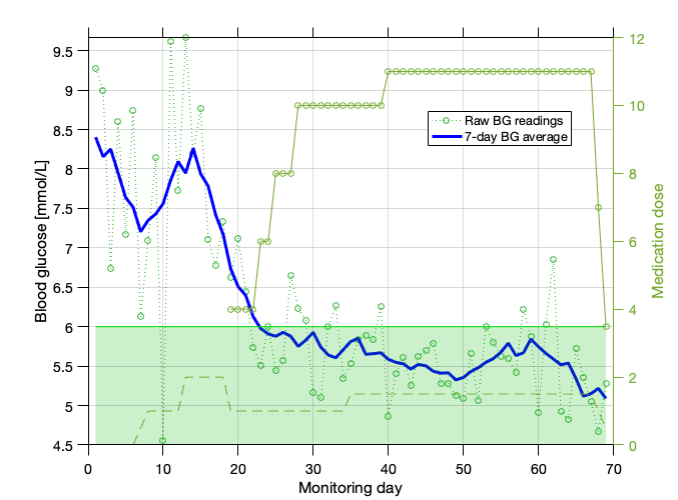
Patient data sample illustrating blood glucose readings, 7-day average blood glucose (BG), medication information, and target range (green shading). Both the dashed and solid circled lines represent medications. In this instance, the use of appropriate medication and dosage allows BG to reach the target range. BG: blood glucose.

Data collected by mHealth platforms (an example is given in Figure 1) will enable the design of algorithms for the identification of women at risk (stratification) and the early detection of required pharmacological treatment (prediction). By using machine learning (ML) to analyze data, it will be possible to provide real-time feedback to women and clinicians and allow the development of decision-supported processes for the titration of medication therapy for hyperglycemia.

This paper aims to describe the application of ML techniques to real-world data collected with an mHealth app to predict future medication events. This study compares how the predictive algorithm performs against standard heuristic techniques currently employed in NHS trusts and provides initial insights on future lines of work that will improve the existing model.

## Methods

### Data Collection

The GDm-Health system (Sensyne Health, plc) was used in research and clinical practice settings, and at the time of writing, it is currently routinely used for clinical practice in 35 NHS trusts.

The GDm-Health system was used to track (1) BG readings and (2) adherence to medications prescribed by health care professionals. Using the GDm-Health app, participants entered their readings, tagged them with information identifying the meal (eg, prebreakfast, postlunch, etc) and recorded information concerning the dose of any medication taken. Whenever enabled, wireless transfer from Bluetooth-enabled BG monitors was used; as an alternative (eg, if there were issues with the wireless transfer or use of a noncompatible meter), manual input of BG readings was also employed.

The analysis was performed on fully anonymized data based on established partnerships with these trusts.

For the analysis in this paper, one source of data was an implementation study that included data from (1) John Radcliffe Hospital, Oxford University Hospitals (OUH) NHS Foundation Trust, and (2) Royal Berkshire Hospital, Royal Berkshire Hospitals (RBH) NHS Foundation Trust.

This implementation study was performed by the Institute of Biomedical Engineering at the University of Oxford and OUH.

Data from the research implementation were collected for the period January 2016 to January 2019 for OUH and September 2014 to September 2019 for RBH.

A second larger set of data was generated by the anonymized data set collected and curated by Sensyne Health, plc, via the GDm-Health system. Anonymization was performed according to guidelines [17] and using a publisher (Privitar, London, United Kingdom).

**Figure 2:**
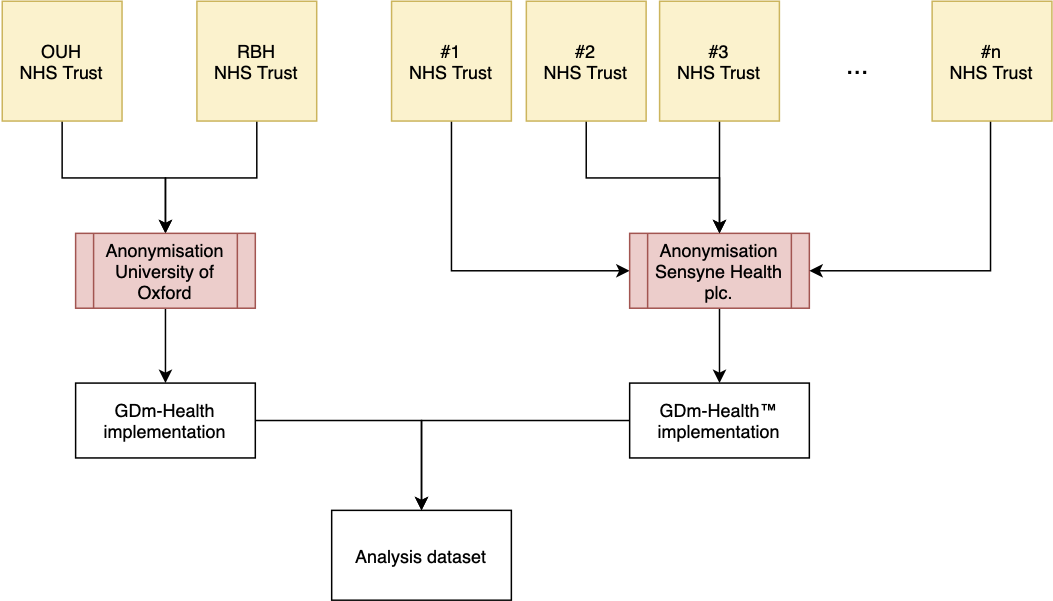
Data collection and anonymization flow. NHS: National Health Service; OUH: Oxford University Hospitals; RBH: Royal Berkshire Hospitals.

Both data sets were analyzed in anonymized form. Anonymization included the removal of identifiable information (eg, given name, family name, NHS number) and transformation of sensitive information (eg, date of birth was transformed into age in years). In both instances (research and production implementation), the systems were used for clinical management of women with GDM; therefore, this analysis corresponds to a retrospective, secondary care–based cohort study of women with GDM using the GDm-Health system (Figure 2 describes the data flow that contributed to the data set used for the analysis).

Pregnant women with GDM used the GDm-Health app to track pre- and postprandial BG. All women with GDM during the monitoring period were included in the analysis. Women with type 1 and type 2 diabetes and Maturity onset diabetes of the young (MODY) were excluded from the analysis.

GDM was diagnosed using a variety of methods, including the International Association of Diabetes and Pregnancy Study Groups criteria [18] and the National Institute for Health and Care Excellence 2010 guidelines [19]. Clinical management included hospital-based follow-up in antenatal clinics and remote monitoring of BG readings using the GDm-Health app. Monitoring and management of patients varied across sites and included management via diet alone, metformin, insulin, or a combination of the above.

### Analysis

To develop an algorithm that could predict women in need of pharmacological treatment, we identified 2 subgroups of interest: the diet–diet group and the diet–drug group. Women belonging to the first group did not have any prescribed medication; therefore, they remained on lifestyle-based therapy throughout their pregnancy. Those belonging to the second group transitioned from lifestyle-based therapy to pharmacological treatment (metformin and/or insulin). We identified patients as belonging to this group when not taking any medication for at least 14 days from the first day of use of the GDm-Health system. If participants initiated pharmacological treatment before the cutoff period (2 weeks), they were excluded from the analysis as they would have initiated pharmacological treatment too early.

**Figure 3:**
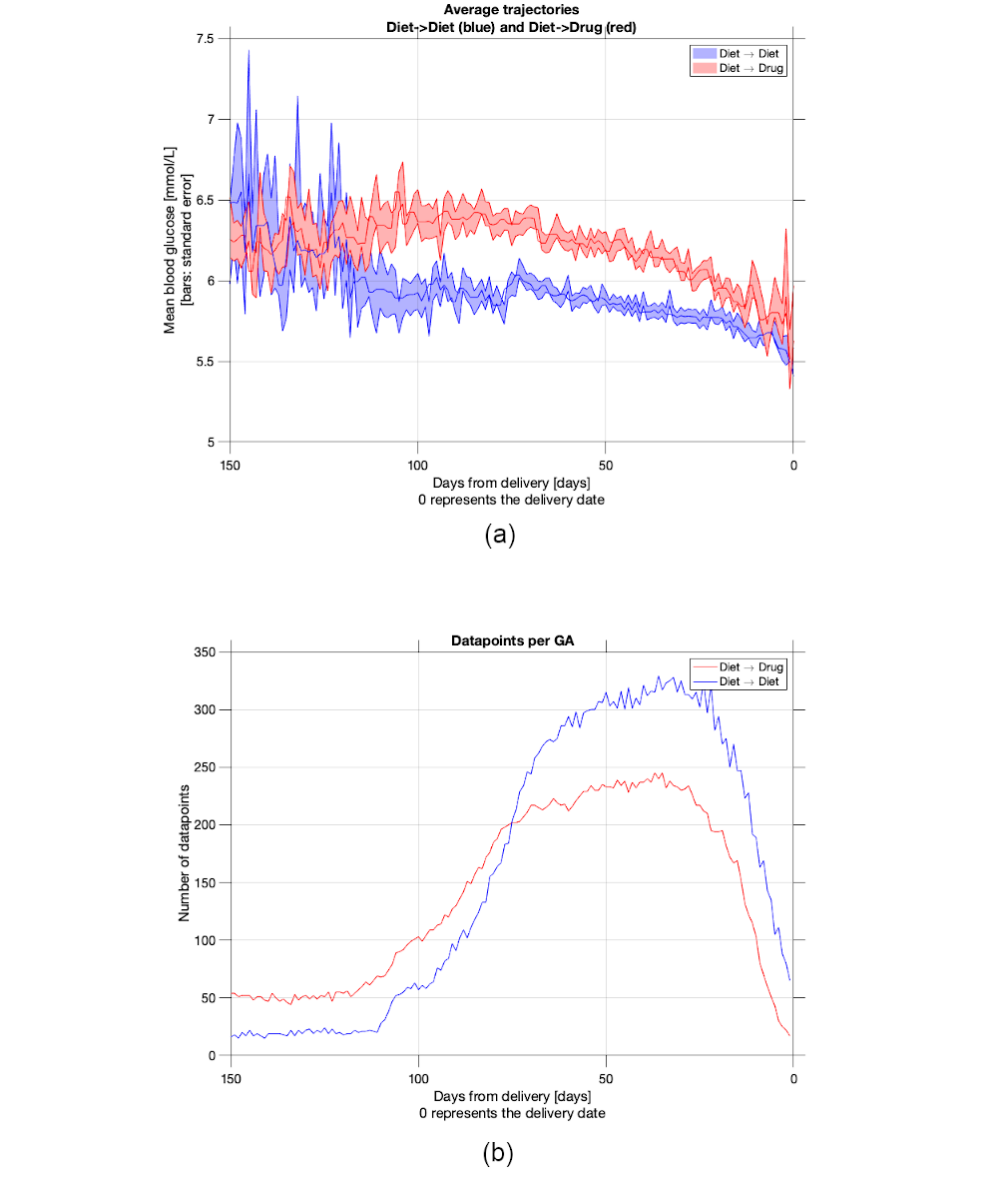
Graph (a) shows the average and SE of blood glucose values per day corresponding to the 2 groups. Graph (b) indicates how many readings per day were used to obtain the average in (a). In both cases, the number of days on the x-axis refers to the number of days to delivery. For the period where the number of readings is high, 100 to 5 days to delivery, which roughly corresponds to the last 3 months, the average of the blood glucose values for the 2 groups is clearly different.

By aligning data from all involved women to their respective delivery date allows us to plot the average BG values regardless of their meal tag and their standard error per day up to delivery. Figure 3 shows the plot of the average BG per day up to the delivery date, and at the same time it shows the number of readings used to compute the statistics of the previous. One can observe how both averages decrease toward the delivery date, suggesting that treatment is successful in both groups. However, the average BG value for those in the diet–drug group was higher than that in the diet–diet group. This result points to the importance of the BG value in deriving features to distinguish women belonging to these 2 groups.

### Clinical Variables

To train a model that is capable of recognizing women belonging to one of the 2 specified groups (diet–diet and diet–drug), we trained an ML algorithm over a set of features (predictors) to be extracted from the training periods associated with the 2 groups.

A list of relevant predictors, informed by clinicians, was drawn up to summarize the monitoring period extracted from the data of each group. Wherever possible, this set of predictors was extended by considering the average and SD of variables over the monitoring period to capture the level and variability of each.

Table 1 lists the set of predictors used for our analysis, together with a detailed explanation of their nature, including the time and the way in which they were recorded. The majority of predictors describe the BG level at different times of the day (eg, prebreakfast, postdinner); 2 identify a consecutive alerting situation (eg, 3 days with high readings at the meal tag in a row), and 2 describe demographics of the patient (age and BMI).

**Table 1:** Description of predictors used in this study.

Feature	How it was expanded	When it was recorded	How it was recorded
Breakfast readings	Mean, SD, min^a^, max^b^, linear regression coefficient	Recordings were made according to the GDM^c^ management plan each participant discussed with their health care professional	BG^d^ data were recorded by participants through the GDm-Health mobile app developed to help mothers-to-be to keep track and manage their BG throughout their pregnancy
Lunch readings	Mean, SD, min, max, linear regression coefficient	Recordings were made according to the GDM management plan each participant discussed with their health care professional	BG data were recorded by participants through the GDm-Health mobile app developed to help mothers-to-be to keep track and manage their BG throughout their pregnancy
Evening meal readings	Mean, SD, min, max, linear regression coefficient	Recordings were made according to the GDM management plan each participant discussed with their health care professional	BG data were recorded by participants through the GDm-Health mobile app developed to help mothers-to-be to keep track and manage their BG throughout their pregnancy
All readings (regardless of prandial tag)	Mean, SD, min, max, linear regression coefficient	Recordings were made according to the GDM management plan each participant discussed with their health care professional	BG data were recorded by participants through the GDm-Health mobile app developed to help mothers-to-be to keep track and manage their BG throughout their pregnancy
Raised 3-day prebreakfast	Variable indicating the number of prebreakfast alerts in a 3-day consecutive period	Recordings were made according to the GDM management plan each participant discussed with their health care professional	BG data were recorded by participants through the GDm-Health mobile app developed to help mothers-to-be to keep track and manage their BG throughout their pregnancy
Raised 3-day postprandial	Variable indicating the number of alerts in a 3-day consecutive period for each postprandial meal	Recordings were made according to the GDM management plan each participant discussed with their health care professional	BG data were recorded by participants through the GDm-Health mobile app developed to help mothers-to-be to keep track and manage their BG throughout their pregnancy
BMI	BMI at the time of booking	Data were recorded at the first encounter with the health care professional	BMI is a derived variable from weight and height. Both these variables were recorded at the time of booking
Age	Age at the time of booking	Data were recorded at the first encounter with the health care professional	Variable derived, for privacy reasons, from the date of birth recorded at booking. Recorded as age in number of months
Ethnicity risk factor	Asian, Chinese, Pakistani, Bangladeshi, and other ethnicities considered at risk for GDM	Data were recorded at the first encounter with the health care professional	Data were manually recorded by the health care professional
Risk factors	Previous GDM, high BMI, family history of diabetes, previous large-for-gestational-age baby, ethnicity, polyhydramnios, glycosuria, macrosomia, missed OGTT^e^, unable to take OGTT, polycystic ovaries	Data were recorded at the first encounter with the health care professional	Data were manually recorded by the health care professional

^a^min: minimum.

^b^max: maximum.

^c^GDM: gestational diabetes mellitus.

^d^BG: blood glucose.

^e^OGTT: oral glucose tolerance test.

Data collected were first analyzed at the population level. BG data distributions were inspected, and sensible (data-driven) thresholds were established to filter out readings that considered outliers. BG monitoring is highly affected by how the test is performed and the experience level of the user. Therefore, inconclusive tests can lead to skewed BG values that are not representative of the real BG levels, and such outliers must be identified and removed to avoid bias in the training data. For the BG level, the 95th percentile of all population data was selected as the cutoff threshold, and values above this level were excluded from the data set as outliers.

As we cannot assume that our analysis would be unaffected by missing data (eg, we cannot guarantee that data are missing entirely at random), variables with missing data were imputed by substituting each missing feature with the values of the population mean for that characteristic.

### Development of an ML Model

**Figure 4:**
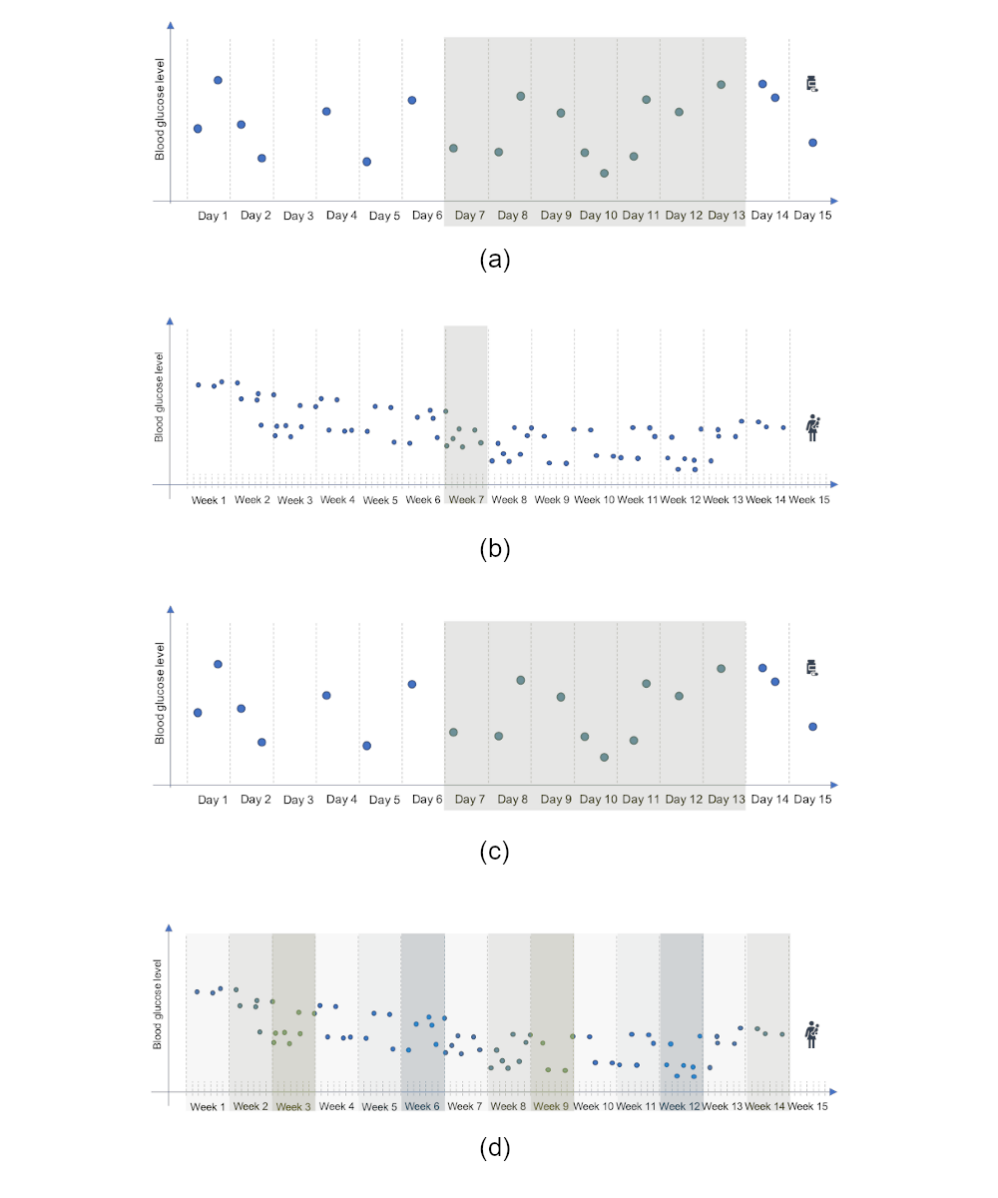
For both (a and b) training and (c and d) validation, summary features are computed on 1 week’s data and a label is assigned according to the group the data belong to (diet–drug and diet–diet). During training, (a) for women with a medication prescription (diet–drug), the week before the first medication dosage is considered for training. A 1-day gap between the training week and the medication event is maintained. (b) For women with no medication prescription (diet–diet), a random week is selected among all those available. During validation, (c) for women in the diet–drug group, we used a similar approach to training and computed the features on the week before the first day of medication (leaving a 1-day gap before the medication event). (d) For women in the diet–diet group, instead, we considered each week available for testing as an independent sample on which to perform a prediction.

Figure 4 shows the learning tasks considered. BG metrics and medication information were available on the GDm-Health platform. Medications are prescribed by health care professionals and are collected and available on the GDm-Health platform as self-reported information by women. Both the type of medication and its dosage were captured in the platform; however, only a binary representation (medication/no medication) was used during the analysis. For the diet–drug group, the training period considered corresponded to the week before the first medication was administered. For the diet–diet group, as it was challenging to identify a clear event and to train over a range of data that represent the whole predelivery monitoring period, a randomly selected week was chosen from all the available ones. For the diet–drug group, we excluded from the training week one day before the start of medication, whereas there was no need for a gap day for the diet–diet group.

Validation was performed similarly to the training for the diet–drug group, that is, based on features computed from the week before the medication event. For the diet–diet group, all available nonoverlapping weeks were independently considered. For each of these, a set of features was generated with the appropriate label for the diet–diet group.

To develop the statistical model, we trained a logistic regression model on training data and tested the output of the training model on the held-out validation data.

We repeated this experiment on 100 different random permutations of the main data set between training and validation data using a 70% training and 30% validation split. At each iteration, to avoid biasing the algorithm toward the overrepresented class (diet–diet), this was randomly downsampled to the number of women in the underrepresented class (diet–drug). The validation set was not downsampled, thereby retaining within it the class imbalance that would be observable in real data. Before training, a lasso feature selection step was performed on the training data and predictors deemed important to this step were selected and used for training the algorithm. The lasso MATLAB (MathWorks) function was used with its *alpha* parameter set to .75 (corresponding to elastic net regression) and using 5-fold cross-validation.

At each iteration, a different set of women would compose the training and testing set, thus training is performed on subjects who do not contribute data to the testing set.

The results of the test were evaluated by computing the receiver operating characteristic curve (ROC), which plots the false-positive rate (FPR) against the true-positive rate (TPR). The AUC was also computed to permit the comparison of different models and to provide a summary of the performance of the algorithm. From the 100 repeated experiments, a summary description of the ROC and AUC was obtained by calculating percentiles at 5%, 50%, and 95%, providing the median ROC curve and CIs at 5% and 95%, respectively.

To compute the ROC curves, risk groups were defined automatically by the perfcurve function (MATLAB 2019a) by varying the value of the decision threshold over the range of values from 0 to 1 produced by the logistic regressor. Finally, comparison with the standard of care was evaluated by visualizing the performance of the current methodology against the ROC curve. The current clinical heuristic states that treatment should be considered if 3 or more consecutive BG readings of the same meal tag are over the designated threshold.

## Results

### Data Description

Data from 12 trusts were collected from women being monitored using the GDm-Health platform during 2019 (Sensyne Health, plc data set) and from the Oxford University research data set between 2014 and 2015.

**Figure 5:**
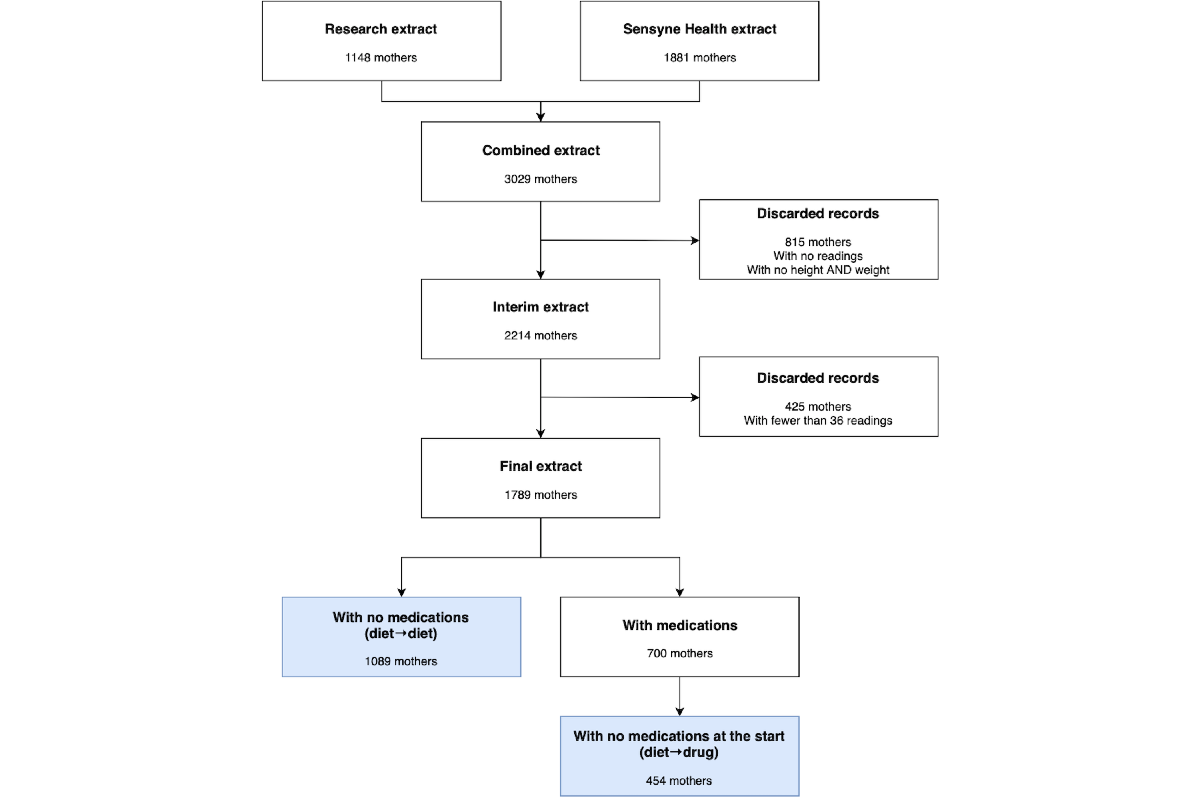
Consort diagram describing the data selection flow. Final groups are identified by the colored boxes.

A total of 3029 women were included in our data set at the time of analysis. After excluding data from women with incomplete demographic information (eg, missing weight and height) and excluding data from women with insufficient BG data (eg, women with fewer than 36 BG readings in the first week of use), data from 1789 women were analyzed. After further reduction and classification into one of the 2 groups of interest for our analysis (diet–diet and diet–drug), the remaining group of women (Figure 5) provided 411,785 BG readings (mean 230, SD 181), of which 160,812 were tagged as breakfast readings, 117,887 as lunch readings, and 133,086 as evening meal readings.

Among the 1789 patients, 39.12% (700/1789) women required pharmacological treatment during their pregnancy, whereas the remaining group was managed only through diet adjustments.

### Analysis

**Figure 6:**
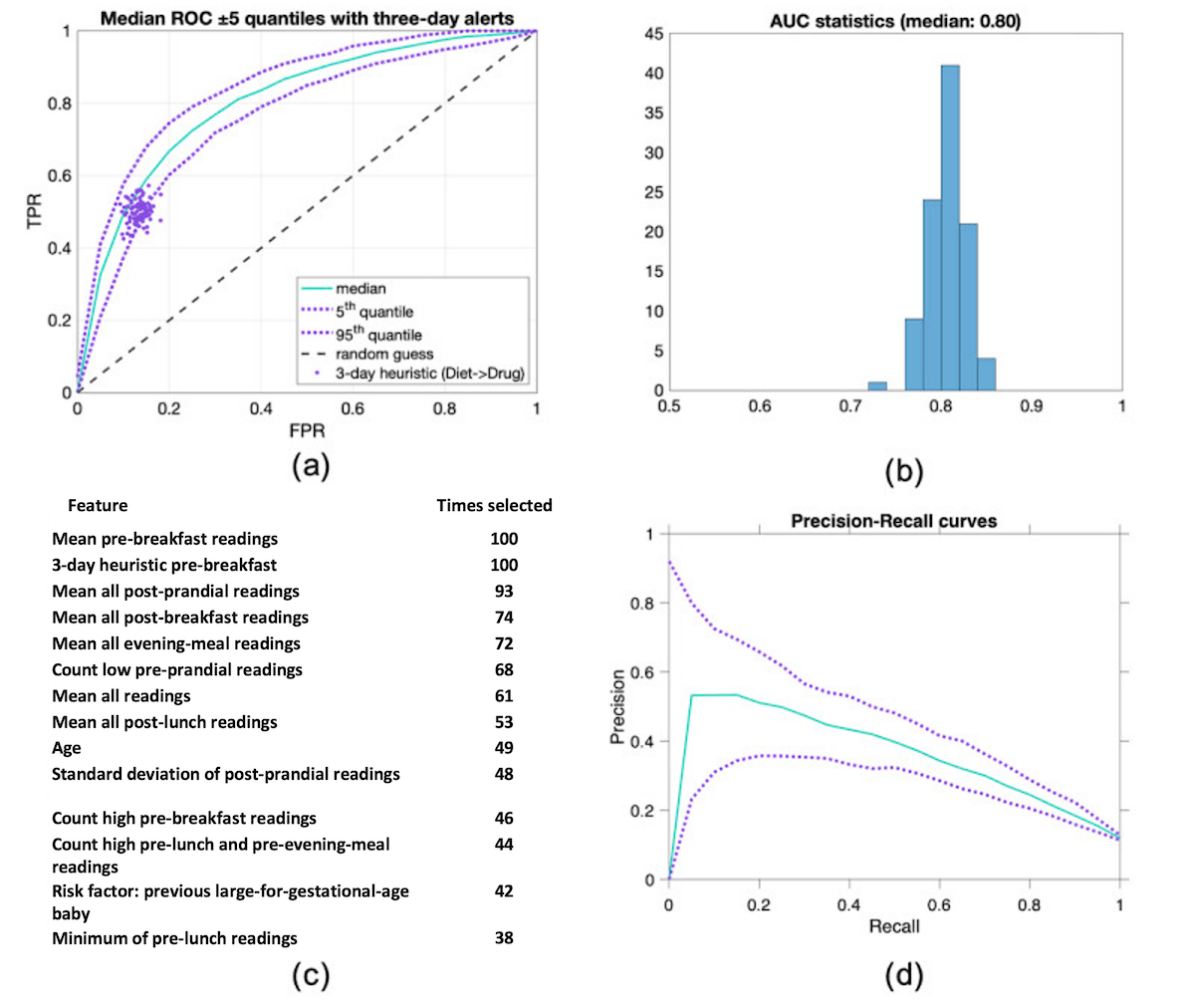
Experimental results. (a) ROC curve depicting the 100 repetitions of the classification experiment (see Figure 3). (b) Histogram showing the distribution of AUC values for all 100 experiments. (c) Ranking of selected variables over 100 repeated experiments. (d) Top ten variables out of 100 repeated experiments. AUC: area under the curve; FPR: false-positive rate; ROC: receiver operating characteristic; TPR: true-positive rate.

The results of the analysis outlined in the Methods section are summarized in Figure 6.

The ROC and AUC results of repeated experiments are shown in Figure 6. Figure 6 also shows the results of the lasso feature selection with the top 14 (top 10 in bold) and summarizes the number of times each was deemed relevant. Figure 6 summarizes the precision-recall curves. Results from the repetition of the validation step are described by the 5%, 50%, and 95% ROC values percentiles; these provide the median ROC curve and corresponding CIs, and the same is done for the precision-recall curves in Figure 6.

The histogram of AUC values drawn from the ROC curves is centered around a median value of 0.80, which supports the potential for clinical evaluation of the proposed algorithmic approach.

**Figure 7:**
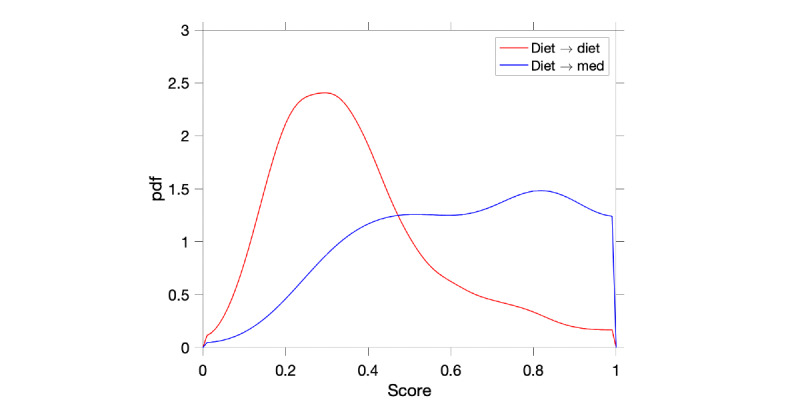
Distribution of scores from a model close to median performance (area under the curve 0.80). The model uses 4 features to perform its function. Although scores for the diet–diet group are clearly distributed on the left-hand side of the graph, the diet–med group presents higher variability.

To evaluate the distribution of scores for a given model, we selected one close to the median performance shown in Figure 6, 0.80 AUC. The selected model operates with 4 features (mean of all BG values, mean of all prebreakfast BG values, max of all postbreakfast BG values, and raised 3-day prebreakfast). Figure 7 shows the distribution of scores for the 2 classes and demonstrates how the model can distribute scores for both classes, although an overlap is presented around the decision boundary. For completeness, additional figures showing the distribution of feature values are shown in the Multimedia Appendix 1 for true positive, true negative, false positive, and false negative.

To compare performance with the standard of care, we evaluated the algorithm against the 3-day heuristic currently used in the trusts, considering that treatment should be considered when 3 or more consecutive BG readings of the same meal tag are over the designated threshold.

As shown in Figure 6, the current heuristic performance is fixed to an area and is nonconfigurable. However, the proposed ML-based approach can make use of a different threshold (eg, to classify differently the score of 0 to 1 output by the logistic regression), thus allowing a change in the operating point of the system and allowing for a larger TPR at the expense of a slightly larger FPR. For example, from Figure 6, we could aim at 80% TPR by increasing our FPR by 20%, thus providing the possibility to the system to identify more women in need of medications, at the added cost of few women who will probably be screened by midwives and identified as false positives.

## Discussion

The increased prevalence of GDM [6] and limited resources available to the NHS [7] pose a problem to the already burdened antenatal care services.

Predicting the need for pharmacological treatment could likely benefit women diagnosed with GDM by improving glycemic control, thereby leading to improved perinatal outcomes and avoiding complications such as large-for-gestational-age newborns or c-sections. Digital health technology such as GDm-Health can provide the real-time monitoring required to collect dense, longitudinal data sets and enable the delivery of clinical decisions quickly and efficiently to patients. Algorithms derived from real-world data obtained from GDm-Health could help midwives to optimize their clinical decision making and allow interventions, including medication, to be delivered earlier.

### Conclusions

In this study, we have used ML on a large, anonymized data set from a population affected by GDM to design an algorithm capable of detecting the need for pharmacological treatment.

The strength of our study lies in the use of a large, multisite, real-world data set to validate our results. Predictors selected by our ML algorithm match most of the predictors included in the state of the art [11-16] and are enhanced by the use of risk factors and other demographic information available as part of routinely collected data by GDm-Health.

The logistic algorithm employed was experimentally tested against LightGBM and Random Forest algorithms. However, when applied to the same features and methodology, these comparator algorithms did not significantly improve AUC performance (both reporting a median of 0.81 AUC).

The aggregated results of the trained logistic regression models achieved an average AUC of 0.80, which is significant to justify future work to evaluate and validate this algorithm in real clinical settings.

Some of the limitations of this study are very common to other mHealth systems, including the challenge of user-reported data such as medication and BG data, which may be inaccurate or missing. However, in the case of GDm-Health data, user retention and user adherence have been very high, with only 4% of profiles being excluded because of complete disengagement with the system (117 women with no readings).

Given the longitudinal nature of this data set (ie, from 2014 to 2019) and the heterogeneous nature of each trust, women at each trust could have been subject to different clinical management processes, adding to the complexity of the ML task.

Nevertheless, we demonstrate that our algorithm predicts the requirement for pharmacological therapy, and we show the superiority of our approach against a heuristic currently employed in clinical settings.

The very likely future introduction of ML algorithms to aid the work of health care professionals and to support patients coping with their conditions requires the validation of the technology using real-world data sets such as the one provided by GDm-Health. We intend to clinically validate the performance of the algorithm further by evaluating its real-time performance on a data set used for clinical operations. To that end, we will first pursue a posthoc analysis on a subset of data not used to design the algorithm and then deploy an implementation of the algorithm alongside GDm-Health to monitor its real-time performance (ie, predictions performed on a daily basis on updated BG daily readings) against decisions performed by health care professionals. Finally, repeated validation and postmarket evaluation strategies will be employed to continuously validate the algorithm against clinical decision making made by health care professionals.

Future work may include new analyses of the GDm-Health data set to include other variables that might identify a change in clinical patient management (eg, including the trust name as a predictor), considering variable lengths of predictive windows (eg, computing features at 2, 3, or 4 weeks before a medication event), or considering the problem as a time-to-event prediction (via Cox proportional hazards, etc).

## Appendix

Multimedia Appendix 1 Error analysis of true positives, true negatives, false positives, and false negatives for a given selected model.

## Abbreviations

AUC: area under the receiver operating characteristic curve
BG: blood glucose
FPR: false-positive rate
GDM: gestational diabetes mellitus
mHealth: mobile health
ML: machine learning
NHS: National Health Service
OUH: Oxford University Hospitals
RBH: Royal Berkshire Hospitals
ROC: receiver operating characteristic curve
TPR: true-positive rate
