# Correction: Toward a Multivariate Prediction Model of Pharmacological Treatment for Women With Gestational Diabetes Mellitus: Algorithm Development and Validation

**DOI:** 10.2196/85415

**Published:** 2025-10-15

**Authors:** Carmelo Velardo, David Clifton, Steven Hamblin, Rabia Khan, Lionel Tarassenko, Lucy Mackillop

**Affiliations:** 1 Sensyne Health, plc Oxford United Kingdom; 2 Department of Engineering Science, University of Oxford Oxford United Kingdom; 3 Oxford University Hospitals NHS Foundation Trust Oxford United Kingdom; 4 Nuffield Department of Women’s Reproductive Health, University of Oxford Oxford United Kingdom

In “Toward a Multivariate Prediction Model of Pharmacological Treatment for Women With Gestational Diabetes Mellitus: Algorithm Development and Validation” [1] the JMIR editors noted one error.

The Figure 6 originally published has been revised due to a typo. The new Figure 6 replacing the originally published one is below.

The previous version of Figure 6 can be found in Multimedia Appendix 1.

The correction will appear in the online version of the paper on the JMIR Publications website, together with the publication of this correction notice. Because this was made after submission to PubMed, PubMed Central, and other full-text repositories, the corrected article has also been resubmitted to those repositories.

**Figure 6:**
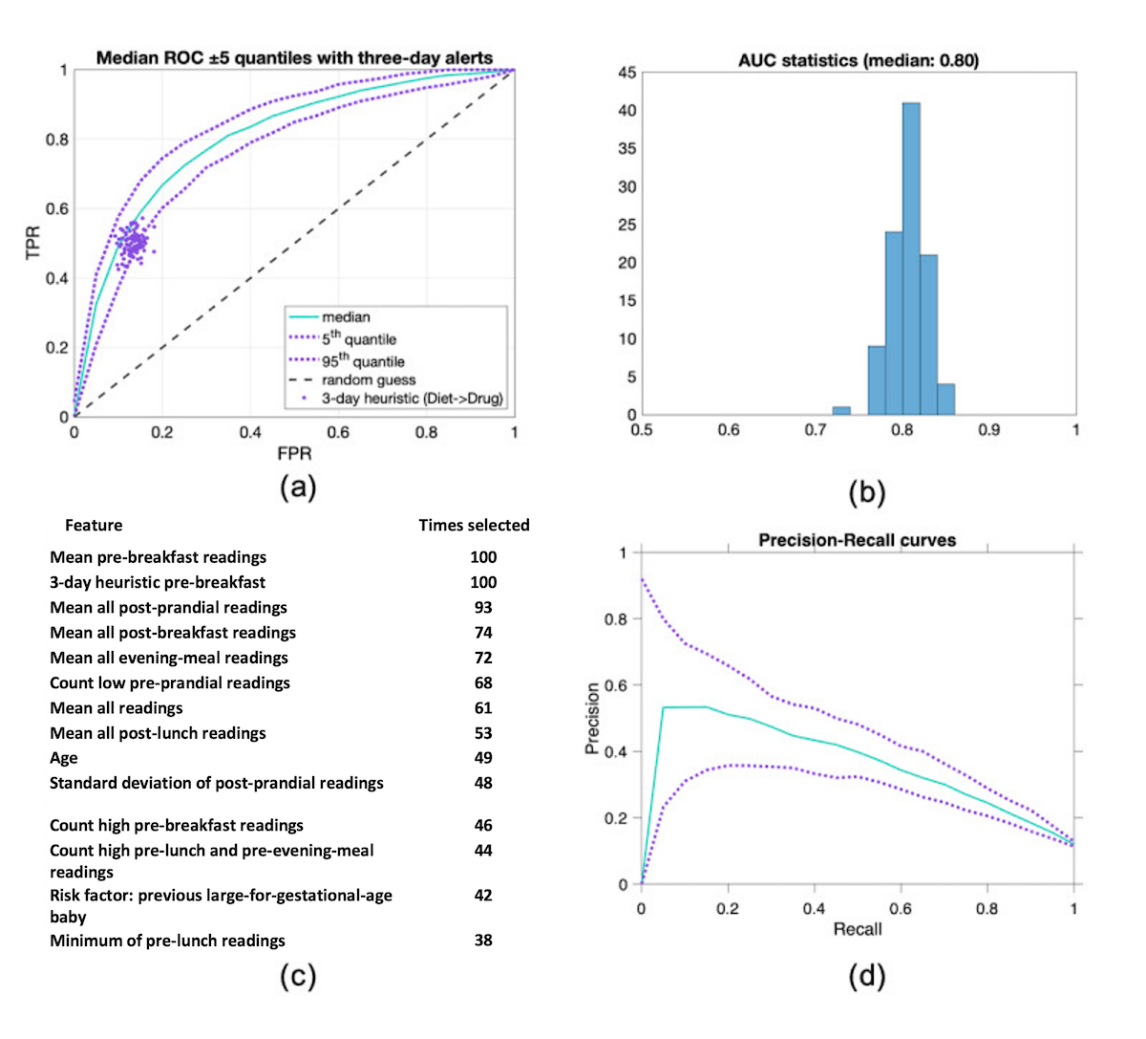
Experimental results. (a) ROC curve depicting the 100 repetitions of the classification experiment (see Figure 3). (b) Histogram showing the distribution of AUC values for all 100 experiments. (c) Ranking of selected variables over 100 repeated experiments. (d) Top ten variables out of 100 repeated experiments. AUC: area under the curve; FPR: false-positive rate; ROC: receiver operating characteristic; TPR: true-positive rate.

## Appendix

Multimedia Appendix 1 Originally published Figure 6.
