# Supplementary material for: Toward a Multivariate Prediction Model of Pharmacological Treatment for Women With Gestational Diabetes Mellitus: Algorithm Development and Validation
Source: J Med Internet Res. 2021 Mar 10;23(3):e21435. doi: 10.2196/21435 (PMC7991989; doi:10.2196/21435)
Supplement: Multimedia Appendix 1 [file jmir_v23i3e21435_app1.docx]

# Appendix

The figures below show the distributions of feature values according to the model selected in Figure 7 of the manuscript following a binarization of the score over a threshold of 0.5.

Scores are shown for the groups of patients categorised as True positives and True negatives (where a classification error does not occur) and False positives and False negatives (where a classification error occurs).

There are clear similarities among different feature distributions although generally the TN and TP seems more separable distributions.

| 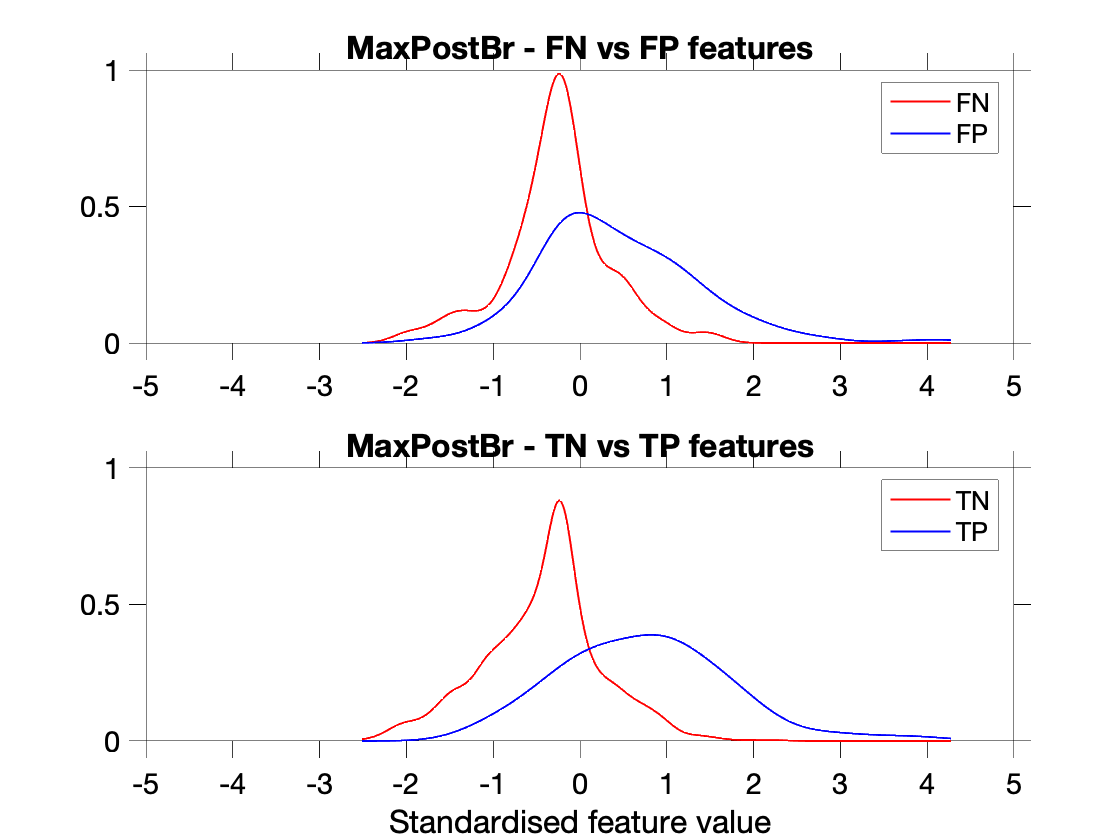 | 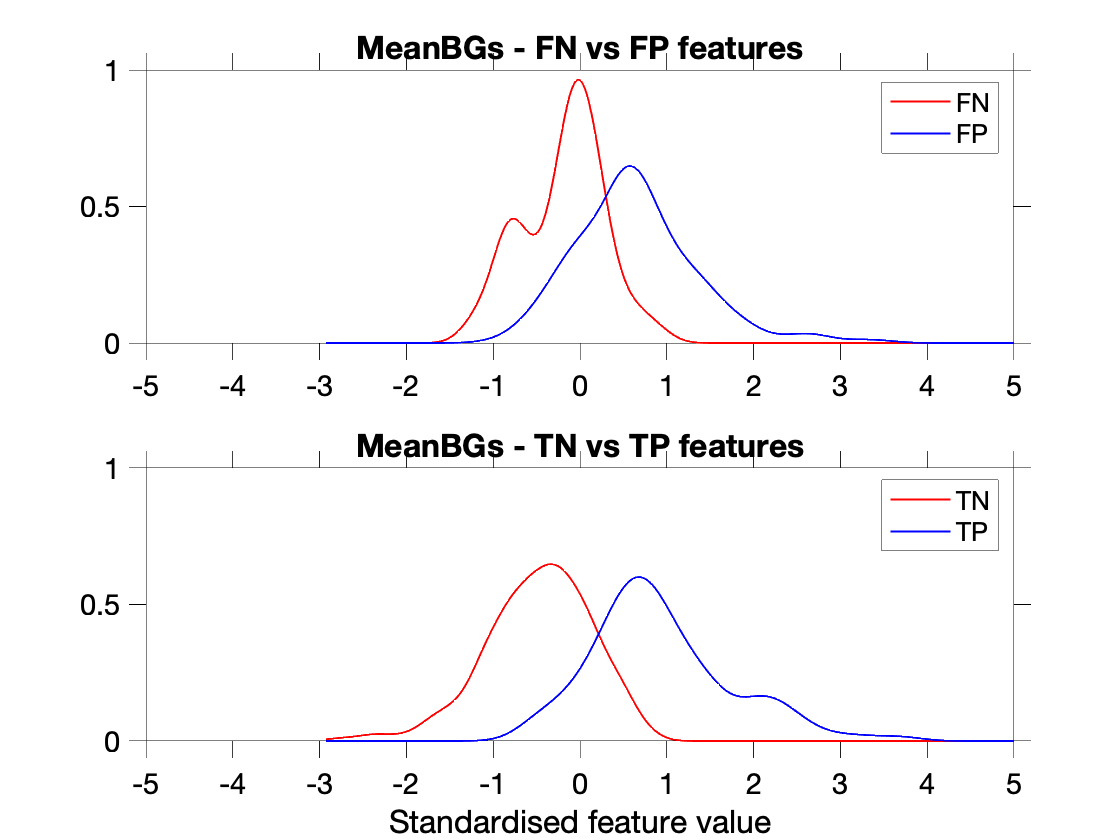 |  |
| --- | --- | --- |
|  |  | |
| 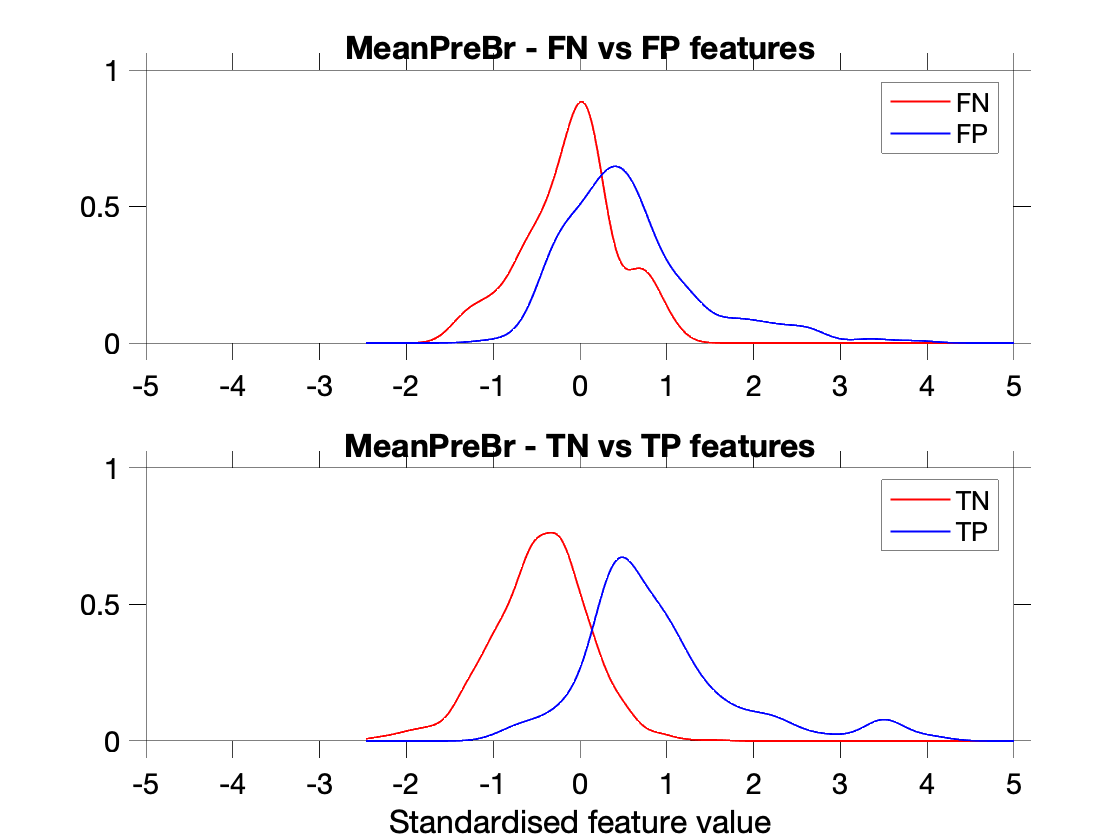 | 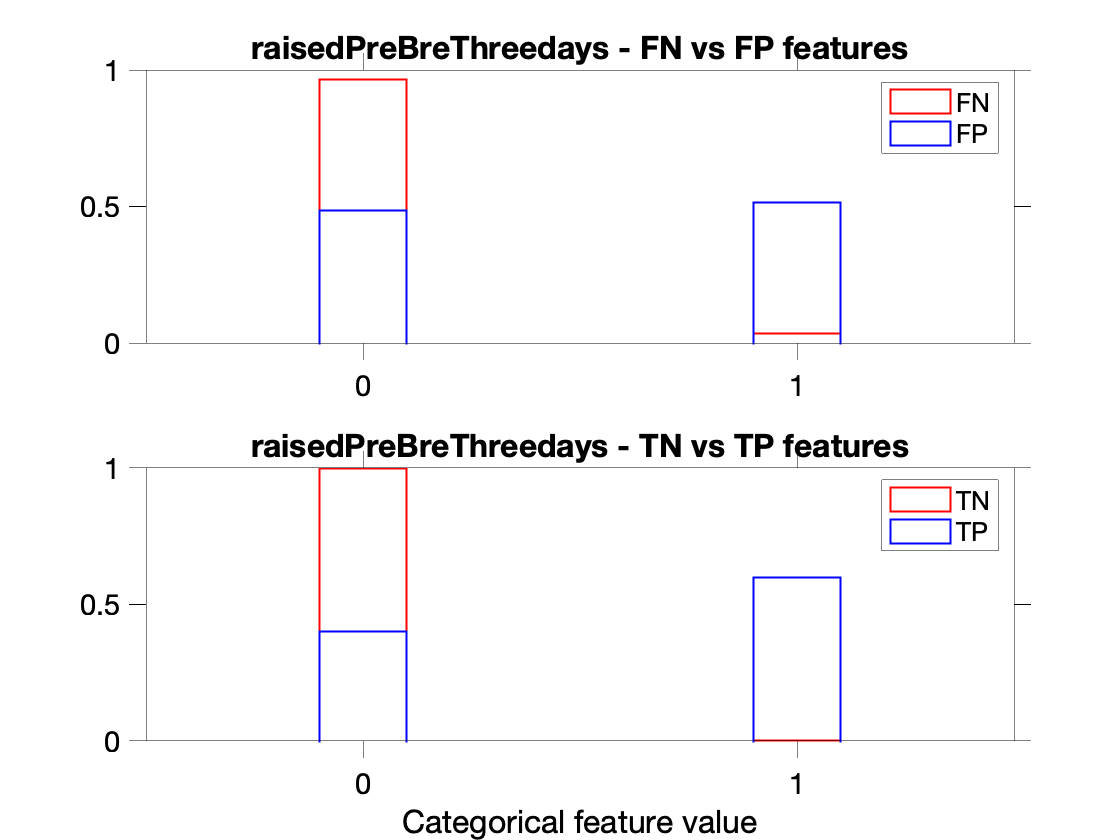 | |
