# Supplementary material for: Correction: Toward a Multivariate Prediction Model of Pharmacological Treatment for Women With Gestational Diabetes Mellitus: Algorithm Development and Validation
Source: J Med Internet Res. 2025 Oct 15;27:e85415. doi: 10.2196/85415 (PMC12572742; doi:10.2196/85415)
Supplement: Multimedia Appendix 1 [file jmir_v27i1e85415_app1.pdf]

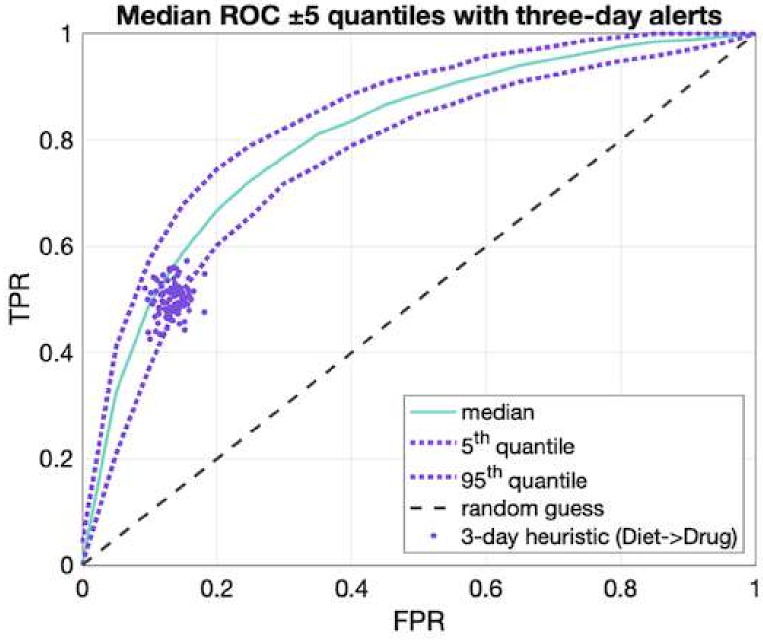

(a)

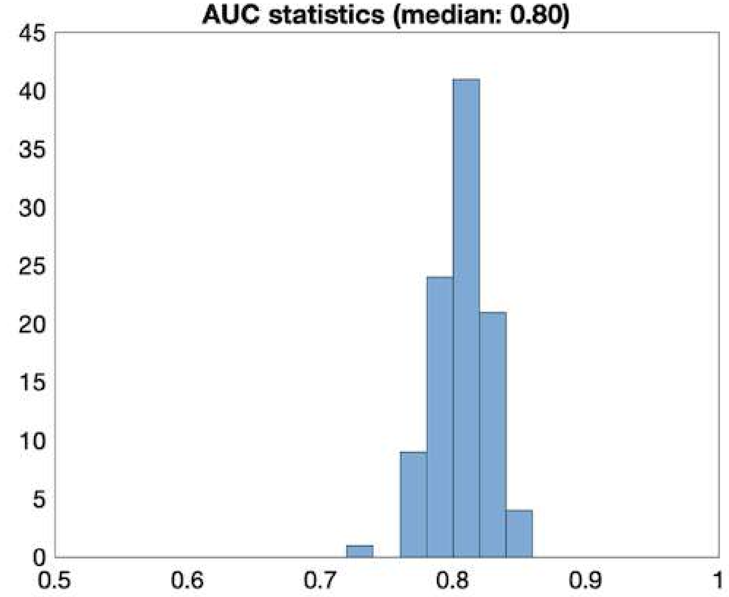

(b)

Feature	Times selected
Mean pre-breakfast readings	100
3-day heuristic pre-breakfast	100
Mean all post-prandial readings	93
Mean all post-brakfast readings	74
Mean all evening-meal readings	72
Count low pre-prandial readings	68
Mean all readings	61
Mean all post-breakfast readings	53
Age	49
Standard deviation of post-prandial readings	48
Count high pre-breakfast readings	46
Count high pre-lunch and pre-evening-meal readings	44
Risk factor: previous large-for-gestational-age baby	42
Minimum of pre-lunch readings	38

(c)

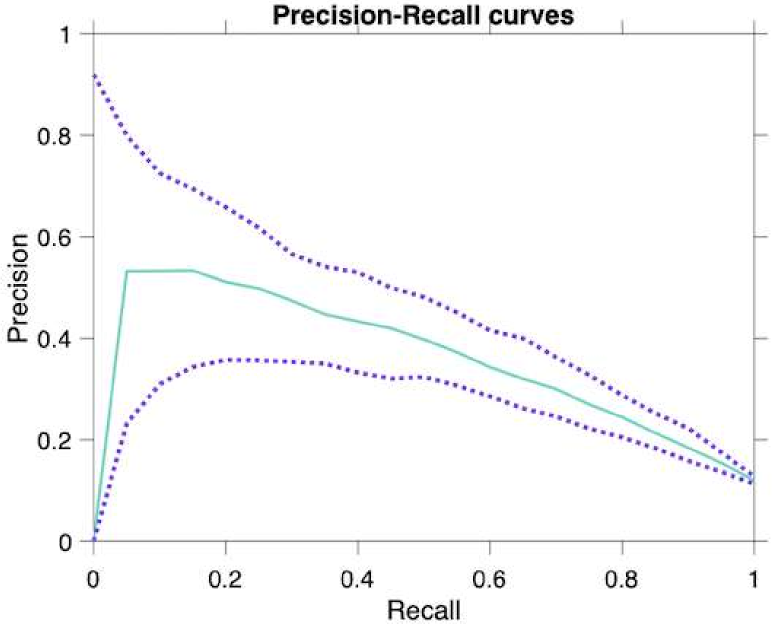

(d)
